# Association of insulin-like growth factor 1 and metabolic parameters with mild subclinical hypothyroidism in obese boys

**DOI:** 10.1038/s41598-025-02084-x

**Published:** 2025-05-16

**Authors:** Jiang Xue, Chen Li, Jing Ju, Shuang Liang

**Affiliations:** Department of Pediatrics, The Second Hospital of Shandong University, 247 Beiyuan Street, Jinan, 250021 China

**Keywords:** Insulin like growth factor 1, Subclinical hypothyroidism, Body Mass Index, Obesity, Boys, Endocrinology, Risk factors

## Abstract

The aim of this study was to investigate the relationship of insulin-like growth factor-1 (IGF-1) and mild subclinical hypothyroidism (MSH) in obese boys and to assess whether the presence of MSH exacerbates cardiovascular risk factors in obesity. This study collected cross-sectional dataset covering 141 obese boys and 47 healthy non-obese boys. The obese group was further subdivided into two groups based on their serum Thyroid Stimulating Hormone (TSH) levels: the MSH group (n = 47) and the non-MSH group (n = 94). The MSH group exhibited significantly lower IGF-1 standard deviation score (IGF-1 SDS) and significantly higher Body Mass Index standard deviation score (BMI SDS) compared to the non-MSH group. Additionally, the MSH group demonstrated elevated triglycerides (TG) and gamma-glutamyl transferase (GGT) levels relative to the non-MSH group, and the incidence of non-alcoholic fatty liver disease (NAFLD) and metabolic syndrome (MS) were also higher in the MSH group than in the non-MSH group. The results of multivariable logistic regression analysis indicated that lower IGF-1 SDS and higher BMI SDS are strongly associated with MSH in obese boys, independently of systolic blood pressure (SBP), diastolic blood pressure (DBP), alanine aminotransferase (ALT), GGT and uric acid. These findings underscore the clinical utility of IGF-1 SDS and BMI SDS as potential biomarkers for identifying MSH-related cardiovascular risks in obese pediatric populations, warranting targeted screening and intervention strategies.

## Introduction

In the past fifty years, the global incidence of childhood obesity has rapidly escalated, becoming a significant worldwide health concern^1,2^. With the rise in obesity rates, numerous studies have focused on the correlation between obesity and subclinical hypothyroidism (SH)^3–5^. SH, also known as isolated hyperthyrotropinemia, is a biochemical condition characterized by serum thyroid-stimulating hormone (TSH) concentrations above the upper limit of the reference range, but normal concentrations of free thyroxine (FT4)^6^. In pediatric populations, SH is further classified based on TSH elevation severity: (1) Mild SH (MSH, grade 1), defined as TSH levels between the upper limit of the age-specific reference range and 9.9 mU/L; and (2) Evident SH (grade 2), defined as TSH ≥ 10 mU/L, with both subtypes maintaining normal FT4 levels. This stratification is critical for clinical decision-making, as the degree of TSH elevation may influence metabolic outcomes and therapeutic interventions^7^. Despite the uncertain underlying mechanisms, numerous studies have confirmed that the prevalence of SH is relatively high among obese children^4,5,8^. However, there is limited research specifically focusing on SH in obese children, especially MSH. Most studies suggest that SH may lead to certain metabolic abnormalities that are aggravated in obese children^9–13^, while a few studies indicate that SH does not exacerbate cardiovascular metabolic risks associated with obesity^14^. There is ongoing debate regarding the impact of MSH on cardiovascular metabolic indicators and the necessity of treatment6^6,7^.

Previous research has indicated a decrease in insulin-like growth factor 1(IGF-1) levels in obese children^15,16^, with reduced IGF-1 levels independently correlated with various cardiovascular risk factors such as nonalcoholic fatty liver disease (NAFLD)^17^, insulin resistance^18^, low high density lipoprotein cholesterol (HDL-C)^15^, and metabolic syndrome(MS)^15^. Akin *et al*^19^ firstly proposed the presence of decreased IGF-1 in adults with SH, a finding further supported by Koyuncu *et al*^20^ in a Turkish cohort. But following studies did not observe any significant differences in IGF-1 in indian girls with or without SH^21^. However, whether there is a change in IGF-1 levels in SH and the potential correlation between IGF-1 and SH in obese boys remains unexplored.

Therefore, this study aims to investigate the potential relationship between MSH, IGF-1, and metabolic consequences in obese boys.

## Materials and methods

### Subjects

This study enrolled participants from the pediatric outpatient clinic of the Second Hospital of Shandong University between December 2022 and December 2023. Written informed consent was obtained from all participants and their parents. The study protocol adhered to the Declaration of Helsinki and was approved by the hospital’s Ethics Committee.

All participants in the obese group were boys aged between 6 and 14 years. The inclusion criterion for the obese group was that the Body Mass Index (BMI) need to be greater than the 95th percentile for age and sex of normal weight individuals^22^. The exclusion criteria for the obese group were: (1) children with organic hypothalamic or pituitary diseases, growth axis disorders, adrenal axis disorders, or gonadal axis disorders; (2) children with TSH levels greater than 9.9mIU/L and autoimmune thyroiditis; (3) children with severe chronic illnesses; (4) children with diabetes or chromosomal abnormalities; (5) children who have used medications that may affect hormone secretion, glucose and lipid metabolism, and body composition. Finally, a total of 141 boys and 47 healthy non-obese boys were selected and included in the study. The obese group was further subdivided into two subgroups based on the presence of MSH^6^: the MSH group (n = 47) and the non-MSH group (n = 94). The control group, comprising 47 boys, was recruited from a pool of non-obese, healthy children and adolescents. Both the obese group and the control group were matched for age, pubertal status, and gender.

MSH was defined as serum TSH is between the upper limit of the reference range and 9.9 mU/L, but FT4 being normal^6^. The normal ranges in our laboratory are TSH 0.51–4.94 mIU/L, free triiodothyronine (FT3) 3.5–6.5 pmol/L and FT4 11.5–22.7 pmol/L. The obese participants were categorized into MSH group and non-MSH group based on their thyroid function test results^6,7^. The diagnosis of NAFLD was estimated with an ultrasound scan, two of the following three abdominal ultrasound findings: (1) diffuse enhancement of liver near field echo (“bright liver”) with stronger echo than kidney; (2) the intrahepatic duct structure is unclear, and (3) the far field echo gradually weakens the liver^23^. The diagnosis of The MS refers to the definition of metabolic syndrome in Chinese children and adolescents^24^.

### Anthropometric measurements

Body weight was measured to the nearest 0.1 kg using a standard electronic scale, and height was measured with a standard height stadiometer (TJ-220S-18, Beideneng, Shanghai, China) to the nearest 0.1 cm. BMI was calculated as weight divided by height squared. The BMI standard deviation score (BMI SDS) was determined based on age- and sex-specific reference values for Chinese children^22^. Pubertal stage was assessed according to Tanner criteria^25^. Blood pressure was assessed using an Audio Intelligent Electronic Sphygmomanometer (HEM-7071, OMRON, China) after a 30-min rest in a supine position in all obese boys. Two measurements of systolic blood pressure (SBP) and diastolic blood pressure (DBP) were taken, and the average of the two measurements was recorded. All measurements were performed by the same pediatric endocrinologist.

### Laboratory measurements

All participants were instructed to fast for 12 h prior to blood tests, which was performed to evaluate endocrine hormone levels, liver function, and other metabolic indicators. The levels of FT3, FT4, and TSH levels were measured using a chemiluminescence analyzer (DXI800, Beckman Coulter, USA). IGF-1 levels were assessed using an automated luminescence tester (Malumi X8, New industry, Shenzhen, China). To mitigate the influence of age, the study utilized the IGF-1 standard deviation score (IGF-1 SDS) to represent the level of IGF-1^26^. The following metabolic parameters were analyzed using an automatic biochemical analyzer (Cobas C702, Roche Diagnostics, Shanghai, China): total cholesterol (TC), high-density lipoprotein cholesterol (HDL-C), low-density lipoprotein cholesterol (LDL-C), triglycerides (TG), fasting plasma glucose (FPG), uric acid, alanine aminotransferase (ALT), aspartate aminotransferase (AST), and gamma-glutamyltransferase (GGT). Fasting insulin levels were measured using a fully automated chemiluminescence analyzer (CobasE602, Roche Diagnostics, Shanghai, China). Insulin resistance was evaluated using the homeostasis model assessment index (HOMA-IR), calculated as fasting insulin multiplied by fasting glucose, divided by 22.5^27^.

### Liver ultrasonography

Liver ultrasonography was performed on subjects following a 12-h fasting period. A 3.5–5 MHz Voluson E8 transducer (GE Healthcare, Tiefenbach, Austria) was used for the ultrasonic scanning of the liver, with all examinations conducted by the same experienced operator.

PASS 11 software was used to calculate the sample size based on the findings of Akin et al.^19^, with IGF-1 as the main reference index. Based on the standard of an alpha of 0.05 (two-sided test) and 90% power, a sample size of 47 was required for the MSH group and a sample size of 94 was needed for the non-MSH group.

### Statistical analysis

Data were presented as mean (± SD) for normally distributed variables or median (interquartile range) for skewed data. Categorical variables were compared using the chi-square test. Differences in continuous variables between two groups were assessed using Students’ t-test for normally distributed data, while the Mann–Whitney U test was employed for skewed variables. The relationship between TSH and other variables was examined using Spearman correlation analysis. Furthermore, partial correlation analysis was applied to clarify the correlation between TSH and other variables after controlling for BMI SDS. Variables that showed statistical significance in the univariate analysis were further included in a multivariate logistic model, with results presented as odds ratios (OR) along with 95% confidence intervals (95% CI). A *P* value of less than 0.05 was considered as statistically significant.

### Patient and public involvement in research

We report no patient or public involvement in the design or implementation of the study.

## Results

### Baseline characteristics of study subjects

The characteristics of MSH, non-MSH obese children and the control group are summarized in Table 1. When comparing the MSH group with the control group, no significant differences were observed in age, gender, FT3, FT4 and FBG levels (*P* > 0.05). The MSH obese group demonstrated significantly higher BMI SDS (*P* < 0.001), elevated TSH levels (*P* < 0.001), and lower IGF-1 SDS (*P* < 0.001). Markers of metabolic dysfunction were substantially worse in MSH participants, including higher TC, TG and LDL-C (*P* < 0.05), lower HDL-C (*P* < 0.001), and greater insulin resistance (HOMA-IR *P* < 0.001) and higher Uric acid (*P* < 0.001). Liver function tests revealed significantly elevated ALT (*P* < 0.001), AST (*P* = 0.004*) and GGT (*P* < 0.001) in the MSH group. These findings demonstrate that obese adolescents with MSH exhibit more severe endocrine-metabolic disturbances across multiple parameters compared to healthy controls.

**Table 1:** Clinical characteristics of MSH, non-MSH obese boys and control group.

Variable	MSH group (n = 47)	Non-MSH group (n = 94)	Control group (n = 47)
Age (yr)	10.00 (8.33–11.00)	10.00 (8.94–11.00)	11.00 (9.00–11.00)
Prepuberty/Puberty, (n)	43/4	90/4	42/5
NAFLD/non-NAFLD, (n)	28/19^#^	35/59	–
MS/non-MS, (n)	16/31^#^	16/78	–
SBP (mmHg)	120.00 (110.00–129.00)^#^	114.00 (108.00–122.00)	–
DBP (mmHg)	80.79 ± 12.52	77.02 ± 7.35	–
FT3 (pmmol/L)	6.37 (5.68–6.83)	6.52 (5.92–7.03)	6.29 (5.79–6.70)
FT4 (pmmol/L)	16.69 (14.33–18.94)	16.56 (14.94–18.29)	16.84 (14.21–18.59)
TSH (μU/mL)	6.36 (5.63–7.68)*^#^	2.76 (2.14–3.57)*	2.01 (1.55–3.01)
IGF-1SDS	− 0.85 ± 1.47*^#^	− 0.11 ± 1.31*	0.37 ± 0.85
BMI SDS	3.20 ± 0.89*^#^	2.85 ± 0.82*	− 0.05 ± 1.06
ALT(U/L)	31.00 (19.00–47.00)*	24.00 (16.00–41.25)*	14.00 (11.00–18.00)
AST(U/L)	25.00 (22.00–39.00)*	26.00 (22.00–34.00)*	22.00 (20.00–26.00)
GGT(U/L)	23.00 (19.00–31.00)*^#^	21.00 (17.00–26.25)*	13.00 (12.00–15.00)
TC (mmol/L)	4.49 ± 0.97*	4.38 ± 0.80*	3.97 ± 0.65
HDL-C (mmol/L)	1.28 ± 0.24*	1.21 ± 0.23*	1.56 ± 0.38
LDL-C (mmol/L)	2.72 ± 0.71*	2.52 ± 0.67*	2.01 ± 0.49
TG (mmol/L)	1.29 (0.99–1.35)*^#^	1.07 (0.76–1.43)*	0.63 (0.48–0.85)
Uric acid (μmol/L)	347.00 (315.00–457.00)*	345.00 (299.75–395.25)*	283.00 (226.00–337.00)
Insulin (μIU/mL)	21.00 (12.74–27.60)*	18.69 (13.55–26.12)*	11.59 (6.43–15.63)
FBG (mmol/L)	5.02 ± 0.58	5.00 ± 0.39	5.05 ± 0.35
HOMA-IR	4.44 (3.02–6.12)*	4.17 (2.75–5.84)*	2.54 (1.45–3.42)

*P < 0.05 versus group; ^#^*P* < 0.05 for MSH group versus non-MSH group.

No significant differences were observed between the non-MSH group and the control group in terms of gender, age, pubertal status, and FBG levels. Within the non-MSH obese group, a notable prevalence of clinical and metabolic abnormalities was evident. Specifically, ALT, AST, GGT, as well as lipid metabolism (TC, LDL-C, and TG), insulin, and HOMA-IR were all significantly elevated compared to the control group (*P* < 0.05). Conversely, HDL-C was significantly lower in the non-MSH obese group (*P* < 0.05). Notably, IGF-1 SDS was significantly lower in the non-MSH obese group compared to the control group (*P* = 0.009). Although thyroid function was within the normal range in both groups, and no statistical differences were observed in FT3 and FT4 levels (all P > 0.05), TSH levels were still significantly higher in the non-MSH group compared to the control group *(P* < 0.001).

Among the 47 enrolled MSH subjects, 28 (59.6%) were identified with NAFLD and 16 (51.6%) exhibited MS. In the 94 enrolled non-MSH subjects, 35 (37.2%) were diagnosed with NAFLD and 16 (17.0%) had MS. The incidence of NAFLD and MS in the MSH group was significantly higher than in the non-MSH group (*P* = 0.019, *P* = 0.032). There were no significant differences in pubertal staging and age between the two groups. As expected, the MSH group had significantly higher level of TSH compared to the non-MSH group (*P* < 0.001), while no statistical differences were observed in FT3 and FT4 levels between the two groups (*P* > 0.05). Moreover, the MSH group exhibited significantly lower IGF-1 SDS (*P* = 0.003) and higher BMI SDS (*P* = 0.016) than the non-MSH group. Additionally, the MSH group had higher SBP, GGT, and TG levels compared to the non-MSH group, while there were no statistical differences in DBP, ALT, AST, TC, HDL-C, LDL-C, uric acid, FBG, insulin, and HOMA-IR between the two groups (all *P* > 0.05).

### Correlations between TSH and other variables

We conducted a bivariate correlation analysis to explore the relationship between TSH and other variables in in MSH and non-MSH groups (Table 2). TSH demonstrated positive associations with BMI SDS (r = 0.272, *P* = 0.001), SBP (r = 0.232, *P* = 0.006), GGT (r = 0.258, *P* = 0.002), TC (r = 0.233, *P* = 0.005), LDL-C (r = 0.291, *P* < 0.001) and TG (r = 0.228, *P* = 0.007). Conversely, TSH was negatively correlated with IGF-1 SDS (r = − 0.207, *P* = 0.014). Moreover, no significant associations were found between TSH and other variables.

**Table 2:** Bivariate correlation analysis between TSH and other variables in MSH and non-MSH groups.

Variable	r	*P*
Age (yr)	− 0.009	0.915
IGF-1SDS	− 0.207	0.014*
BMI SDS	0.272	0.001*
SBP (mmHg)	0.232	0.006*
DBP (mmHg)	0.158	0.061
ALT(U/L)	0.160	0.058
AST(U/L)	0.030	0.723
GGT(U/L)	0.258	0.002*
TC (mmol/L)	0.233	0.005*
HDL-C (mmol/L)	0.154	0.068
LDL-C (mmol/L)	0.291	< 0.001*
TG (mmol/L)	0.228	0.007*
Uric acid (μmol/L)	0.110	0.194
Insulin (μIU/mL)	0.134	0.113
FBG (mmol/L)	− 0.057	0.499
HOMA-IR	0.131	0.122

*P < 0.05.

### Correlations between TSH and other variables after control BMI SDS

To mitigate the confounding influence of BMI SDS on the statistical results, we performed a partial correlation analysis to isolate the impact of BMI SDS on the statistical results. After controlling for BMI SDS, in obese boys, TSH remained positively correlated with SBP (r = 0.190, *P* = 0.025), TC (r = 0.169, *P* = 0.045), and LDL-C (r = 0.211,* P* = 0.012), whereas it exhibited a negative correlation with IGF-1 SDS (r = − 0.229, *P* = 0.007). But after controlling for BMI SDS, the correlations between TSH and both GGT (r = 0.161, *P* = 0.057) and TG (r = − 0.048, *P* = 0.570) became insignificant.

### Univariate and multivariable risk factor analyses for MSH

To identify risk factors associated with MSH, we performed both univariate and multivariate logistic regression analyses. The univariate analysis revealed significant associations between MSH and the following variables: IGF-1 SDS, BMI SDS, SBP, DBP, ALT, GGT, and Uric acid, all of which were included in the final multivariate model (Table 3). The results of the multivariable logistic regression analysis revealed that the following factors were associated with MSH independent of the other cardiovascular risk factors (Table 4): IGF-1 SDS (OR: 0.639, 95% CI 0.476–0.858, *P* = 0.003) and BMI SDS (OR: 1.795, 95% CI 1.135–2.840, *P* = 0.012). These findings indicate that BMI SDS served as an independent risk factor for MSH, while IGF-1 SDS was found to be a protective factor for MSH in obese boys.

**Table 3:** Variables independently associated with MSH in Univariate analysis.

Variables	OR (95% CI)	*P* value
IGF-1SDS	0.679 (0.520–0.888)	0.005*
BMI SDS	1.661 (1.088–1.536)	0.019*
SBP (mmHg)	1.041 (1.008–1.015)	0.016*
DBP (mmHg)	1.044 (1.004–1.085)	0.030*
ALT(U/L)	1.011 (1.001–1.021)	0.039*
GGT(U/L)	1.031 (1.005–1.057)	0.019*
Uric acid (μmol/L)	1.006 (1.002–1.011)	0.007*

*P < 0.05.

**Table 4:** Variables independently associated with MSH in Multivariate analysis.

Variables	OR (95% CI)	*P* value
IGF-1SDS	0.658 (0.489–0.885)	0.006*
BMI SDS	1.686(1.058–2.686)	0.028*

*P < 0.05.

## Discussion

In the study, we observed that BMI SDS emerged as an independent risk factor for MSH, while IGF-1 SDS served as an independent protective factor against MSH in obese boys. In addition, the MSH group exhibited significantly higher BMI SDS, SBP, GGT and TG levels compared to the non-MSH group. The incidence of NAFLD and MS were also higher in the MSH group than in the non-MSH group.

We also provided evidences that IGF-1 SDS was significantly associated with MSH in obese boys. First, lower IGF-1 SDS levels were observed in the participants with MSH compare with non-MSH group among obese boys. Second, a negative correlation between TSH and IGF-1 SDS was identified in the bivariate correlation analysis. Third, the results of multivariable logistic regression analysis further revealed that IGF-1 SDS was an independent protective factor for resisting MSH.

The mechanism underlying decreased IGF-1 levels in obese boys with MSH remains unclear; however, several possible explanations can be proposed: first, studies have confirmed that the growth hormone (GH) releasing hormone receptor in the anterior pituitary lobe of rats with hypothyroidism is downregulated, leading to a decrease in GH production. The insufficient secretion of GH mediates the decrease in IGF-1 secretion^28,29^. Clinical studies in children and adults have also yielded consistent results^19,30^. Second, the elevation of leptin related to obesity can regulate TSH secretion^31^. There is a clear positive correlation between leptin and TSH^8^. On the other hand, leptin and IGF-1 are highly correlated and region-specific in adipose tissue of growing rats^32^, suggesting that leptin may be involved in regulating changes in IGF-1 in SH. Third, another possible explanation for the connection between IGF-1 and SH is driven by chronic inflammation. On the one hand, the secretion of inflammatory cytokines in obese adipose tissue can inhibit the secretion of IGF-1^33^, while high levels of thyroid stimulating hormone increase the expression of serum inflammatory markers^34^. Finally, not all relationships between thyroid hormones and IGF-1 are mediated through GH expression and release. There may be other mechanisms independent of GH that are involved^35,36^.

As demonstrated in previous studies^15–18^, reduced IGF-1 levels are independently associated with multiple cardiovascular risk factors, including NAFLD, insulin resistance, dyslipidemia (particularly low HDL-C) and metabolic syndrome. Our findings of lower IGF-1 levels in obese boys with SH suggest that these metabolic associations may also apply to SH in pediatric obesity. Given the established links between decreased IGF-1 and metabolic dysfunction, our results further support the potential utility of IGF-1 as a biomarker for metabolic health assessment in obese youth. Future longitudinal studies should investigate whether IGF-1 trajectories predict the progression of metabolic comorbidities in this population, which could help guide targeted interventions to mitigate obesity-related complications.

Previous studies focusing on the impact of SH on lipid metabolism in obese children have yielded inconsistent findings. Some studies identified changes in lipid profiles in SH patients^8,9,11,37^, while others reported no significant differences^13^. The inconsistent findings of these studies may be attributed to variations in study design, study populations, and individual variations among participants. This study also focused on the influence of MSH on lipid metabolism. The results demonstrated that TG levels were significantly higher in the SH group compared to the non-MSH group, while other lipid metabolism indicators showed no statistically significant differences between the two groups. In the correlation analysis between TSH and lipid metabolism indicators, after controlling for the influence of BMI SDS, a significant correlation was still observed between TSH and TC as well as LDL-C. The specific mechanism underlying the alterations in TSH and lipoproteins remains unclear, but it may be related to the effect of elevated TSH on lipoprotein transfer and the decrease in lipoprotein lipase activity^38^. The results of this study are consistent with prior findings presented by Krause et al.^8^ and Di Sessa et al.^37^. Interestingly, although there were no statistically significant differences in other lipid metabolism indicators between the two groups, the correlation between TSH and most lipid metabolism indicators suggests that children with SH may develop abnormalities in lipid metabolism during long-term follow-up. This aligns with finding of previous studies^6^, highlighting the importance of long-term monitoring of lipid metabolism indicators in obese children with SH.

Previous studies have extensively explored the correlation between hypothyroidism and NAFLD in adults^39–41^. Similarly, Di Sessa et al. and Choi et al. have reported a clear association between SH and NAFLD in obese children^37,42^, which is consistent with the findings of our study. GGT serves as an important biomarker for advanced fibrosis in NAFLD^43^ and plays a pivotal role in the development of NAFLD, making it a crucial parameter for clinical monitoring. Our study found that in obese boys, the GGT levels were significantly higher in the MSH group compared to the non-MSH group, while there were no statistical differences in ALT and AST levels between the two groups. This finding is in line with the observations made by Ozalp et al^11^, who reported no significant changes in ALT and AST levels in obese children with SH. However, unlike Ozalp et al^11^, our study included GGT as a biomarker and identified a notable correlation between TSH and GGT in obese children. Interestingly, after adjusting for BMI SDS, the correlation between TSH and GGT became insignificant (r = 0.161, *P* = 0.057). This suggests that the correlation between TSH and GGT may be influenced by variations in body weight, implying that body weight could be could be a key mediator in this association.

While insulin resistance represents a central feature of obesity, its association with SH remains controversial. In our cohort of obese children, we observed no significant differences in insulin levels or HOMA-IR between MSH and non-MSH groups. This finding aligns with a cross-sectional study of Korean obese children with SH^5^, but contrasts with a Brazilian study demonstrating an independent association between TSH and HOMA-IR in overweight/obese adolescents^13^. The relationship between TSH and insulin resistance remains debated even among euthyroid children^44–47^. These discrepant findings may be attributed to several factors: (1) variations in study populations regarding age, pubertal status, and degree of obesity; (2) differences in ethnic susceptibility to metabolic dysregulation; (3) potential threshold effects of TSH on insulin sensitivity; and (4) methodological variations in assessing insulin resistance. These findings underscore the need for standardized assessments of thyroid-metabolic axes in diverse cohorts to clarify these associations and their clinical implications.

This study also has several potential limitations. Firstly, given its cross-sectional design, it is imperative to conduct longitudinal studies to establish a causal relationship between TSH and IGF-1. Second, while the study exclusively focused on boys to avoid confounding effects of gender differences on the results, this limitation highlights the need for further research to explore potential gender-specific variations in the TSH-IGF-1 relationship. Another limitation of this study is the relatively small sample size of the healthy control group, primarily constrained by practical challenges in pediatric subject recruitment. These challenges include lower parental acceptance rates for blood sampling in asymptomatic children and stringent ethical review requirements for non-therapeutic research involving minors. Lastly, this study not clarified whether levothyroxine treatment could reverse the decreased IGF-1 levels, which encourage further research to further confirm this question.

## Conclusion

In conclusion, we have identified a robust correlation relationship between low IGF-1 SDS, higher BMI SDS and MSH in obese boys, independent of other cardiovascular risk factors. Additionally, MSH in obese boys appears to be associated with subtle atherogenic abnormalities. These findings holds significance for the clinical management of MSH, highlighting the importance of monitoring alterations in IGF-1, BMI, and other metabolic indicators in obese boys presenting with concurrent MSH. Importantly, the potential benefits of initiating levothyroxine treatment in improving metabolic abnormalities associated with SH warrant further investigation. Additionally, elucidating the underlying pathophysiological mechanisms connecting IGF-1 and MSH remains a key area for future research.

## Data Availability

The data and materials presented in this study are available on request from the corresponding author.
